# A unified framework for federated discovery, interrogation, and analysis using the GAAIN and AD Workbench platforms: A case study using the BLSA cohort

**DOI:** 10.1002/alz.095835

**Published:** 2025-01-09

**Authors:** Ioannis Pappas, Cally Xiao, Michael E. Griswold, Mukta Phatak, Arthur W. Toga

**Affiliations:** ^1^ Laboratory of Neuro Imaging, Stevens Neuroimaging and Informatics Institute, Keck School of Medicine, University of Southern California, Los Angeles, CA USA; ^2^ University of Mississippi Medical Center, The MIND Center, Jackson, MS USA; ^3^ Alzheimer’s Disease Data Initiative, CA USA; ^4^ Laboratory of Neuro Imaging, USC Stevens Neuroimaging and Informatics Institute, Keck School of Medicine, University of Southern California, Los Angeles, CA USA

## Abstract

**Background:**

There is a consensus in the Alzheimer’s disease and related dementias (ADRD) community that data sharing can accelerate science leading to better biomarkers, treatments and preventative measures. However, the ADRD community faces extensive challenges when it comes to sharing of complex biomarker data due to heavy‐handed data sharing mandates and various types of restrictions.

**Methods:**

The Global Alzheimer’s Association Interactive Network (GAAIN) and the Alzheimer’s Disease (AD) Data Initiative have pioneered methods in federated data sharing and learning methodologies by allowing data partners to share their data while protecting their data ownership rights. Users can access, interrogate, and analyze data in a way that is functionally equivalent to raw data access. These two platforms have made a strategic partnership to enhance data interoperability and analysis by using powerful data interrogation and analysis techniques that are provided to the users for free.

**Results:**

For this presentation we will demonstrate data discovery, interrogation, and analysis using the GAAIN platform and the AD Workbench, which is the AD Data Initiative’s cloud‐based platform. We will showcase an example of data analysis using the Baltimore Longitudinal Study of Aging (BLSA), America’s longest‐running scientific study of human aging and related cognitive and physical changes. We will use GAAIN and the AD Workbench for data discovery and advanced statistical modeling for the purposes of understanding risk factors for dementia in the BLSA cohort.

**Conclusions:**

We show an end‐to‐end pipeline of data discovery, interrogation, and analysis using the respective GAAIN and AD workbench tools. We are hoping that similar workflows can be incorporated by stakeholders thus increasing data and tool sharing among the ADRD community.

